# Comparative adaptations of high-tolerant species and broccoli cultivars to salinity stress during germination and early development stages

**DOI:** 10.1186/s12870-025-06723-3

**Published:** 2025-05-27

**Authors:** Angel Almagro-Lopez, Juan Nicolas-Espinosa, Jose M. Mulet, Micaela Carvajal

**Affiliations:** 1 Aquaporins Group, Centro de Edafologia y Biologia Aplicada del Segura, CEBAS-CSIC, Campus Universitario de Espinardo-25, Murcia, E-30100 Spain; 2 Instituto de Biología Molecular y Celular de Plantas (IBMCP), Universitat Politècnica de València- Consejo Superior de Investigaciones Científicas, Valencia, 46022 Spain

**Keywords:** Salinity, Germination, Broccoli, ATP, Development, Antioxidant

## Abstract

Salinity imposes significant physiological and biochemical challenges on plants, disrupting key processes such as germination, involving growth, and water balance. Under saline conditions, plants activate various defense mechanisms to mitigate salinity-induced damage. While many of these mechanisms are well-characterized in mature plants, their role during germination and early seed development remains largely unexplored. In this work, we studied four pre-commercial broccoli (*Brassica oleracea* L. var. italica) cultivars previously selected for their enhanced salinity tolerance and compared to the high tolerant *Eruca vesicaria subsp. sativa*. The results provide insights into key mechanisms involved in salinity tolerance, including osmotic potential regulation, mineral homeostasis, antioxidant enzymatic activity and ATP concentration. The ATP availability and utilization emerged as critical determinants of the stress response profiles of the seeds during germination. Notably, the BQ1 cultivar demonstrated the most efficient ATP utilization, suggesting a broader, more sustained, and effective response under saline conditions. These findings highlight ATP as a crucial factor in salinity tolerance during early seeds development.

**Supplementary Information:**

The online version contains supplementary material available at 10.1186/s12870-025-06723-3.

## Introduction

Salinity is one of the largest global challenges in arid and semi-arid regions, severely affecting agricultural production [1]. Salinity affects approximately 20% of the total cultivated land, and 33% of the irrigated agricultural lands worldwide [2, 3], a situation expected to worsen and accelerate by 2050, due to climate change. Moreover, factors such as rising sea levels lead to seawater intrusion into aquifers due to excessive groundwater extraction in dry regions [4].

Salinity stress refers to the high concentration of salt ions in the soil, especially Na^+^ and Cl^−^ [5], which leads to osmotic stress and ionic toxicity [6]. Osmotic stress occurs within first minutes of salt accumulation in the root area. Excessive deposits of salt reduces the osmotic potential of the soil thereby reducing water absorption of the roots resulting in reduced growth rate [4, 5]. Ionic stress, on the other hand, is observed in the shoots from a few minutes to few hours due to prolonged ion accumulation, particularly Na+, causing tissue toxicity. Excessive salt uptake via transpiration can also result in cellular damage within transpiring leaves, potentially causing further reductions in growth and, in severe cases, plant death [4, 5].

Numerous studies have been published on the effects of salinity on plant species primarily focus on sprouts and adult plants [7, 8]. While these studies are valuable, it is equally important to investigate the impact of salinity during the first developmental stages of the plant. Salt stress affects key physiological and biochemical processes, including germination, growth, photosynthesis, water relations, nutrient homeostasis, oxidative stress management, and yield [9]. Seed germination is one of the most fundamental and vital stages in the growth cycle of a plant [4]. Germination is particularly important among plant species cultivated for sprout production, which are extensively utilized as food. Broccoli (*Brassica oleracea L. var. italica*) has gained popularity due to its excellent nutritional and biochemical properties [10]. Recently, the importance of broccoli sprouts as a nutrient source has been studied. Broccoli sprouts are considered to be highly nutritious food compared to mature inflorescence because of their significantly higher levels of bioactive compounds such as glucosinolates and phenolic compounds [11].

Ion toxicity interferes with enzyme activity, damages membranes, and disrupts metabolic pathways, causing increased production of reactive oxygen species (ROS) such as superoxide radicals (O₂⁻), hydrogen peroxide (H₂O₂), and hydroxyl radicals (OH-), which are harmful to different development stages of the plant, including germination [12]. ROS are highly reactive molecules that damage proteins, lipids, DNA, and other cellular components when its presence is beyond the threshold level [13]. Oxidative damage is associated with increased levels of malondialdehyde (MDA), a hallmark of membrane damage at a cellular level [14]. To prevent oxidative damage caused by ROS, plants have developed a complex antioxidant system. The primary components of this system include ascorbate and glutathione in addition to enzymes such as catalase (CAT), ascorbate peroxidase (APX) and glutathione reductase (GR) [15]. The functionality of this complex antioxidant network is dependent on ascorbate and glutathione pools, which are directly related to NADPH and ATP levels in the cell [16]. This is even more critical during germination under salinity, as energy levels are directly related to ATP reservoirs within the seed.

Similarly, osmotic stress affects the water uptake process during seed imbibition, as reported by Khan et al. [17], while ion toxicity disrupts the enzymatic activities associated with nucleic acid and protein metabolism [18, 19]. Additionally, salinity interferes with metabolic equilibrium [20] resulting to an impaired mobilization and utilization of seed reserves [21]. Under salinity stress, efficient mechanisms related to vacuole storage of excess Na⁺ ions are essential [22]. The majority of these mechanisms are ATP-dependent, highlighting the critical role of intracellular energy levels in maintaining ion homeostasis [23].

Under salt stress conditions, various strategies have been described to enhance plant growth and yield. These strategies underline the critical need of developing suitable cultivars with improved crop performance and germination under salt stress [24]. Comparative studies of physiological salinity responses among varieties provide valuable insights into differences in growth patterns and salt tolerance levels [25, 26]. However, the investigation regarding the salt stress resilience of different genotypes across the different developmental stages has been proven to be challenging [25, 27]. Notably, the response to salinity at germination and seedling stage, as both stages are highly susceptible to salt stress. In this context, an investigation of the diverse responses exhibited by salt-tolerant species during germination can facilitate the identification of resilience related traits at the early developmental stages. These traits can then be used as focal points for breeding programs aimed at developing new and more resilient cultivars [28].

In this study, we investigate the effect of salinity on seed germination by analyzing the germination rate, the mineral nutrient composition and the antioxidant metabolism (enzymes and lipid peroxidation) in relation with ATP availability which is an underexplored parameter in germination studies regarding salinity stress. For that, four pre-commercial broccoli cultivars in comparison to another *Brassicaceae* species, *Eruca vesicaria subsp. Sativa* (*E. vesicaria*) that is known for its salt-tolerance [29, 30] were evaluated in the current study. *E. vesicaria* specie was selected due to availability of seed stocks and for its optimal biomass production. This comparative study can provide novel identification of early-stage metabolic and oxidative stress responses potentially related to salt adaption strategies. This methodology enables the assessment of the salt stress response of the cultivars and the various adaptive strategies utilized in response to salt stress.

## Materials and methods

### Germination and growth conditions

Seeds of the four broccoli cultivars (BG1, BH1, BX1 and BQ1) were provided by Sakata (SAKATA SEED IBÉRICA SLU) while *E. vesicaria* seeds were purchased from CANTUESO (CANTUESO Natural Seeds). To ensure an antiseptic environment, 25 seeds from each species and cultivar were sterilized for 10 min in a 1:1 water-sodium hypochlorite solution. Following disinfection, the seeds were rinsed with distilled water and sown on square Petri dishes (12.5 × 12.5 cm) lined with two layers of filter paper and containing 7 ml of a respective salt solution (50 mM, 100 mM, or 150 mM NaCl) or distilled water (control). Each treatment for the broccoli cultivars and *E. vesicaria* had four replicates with a total of 100 seeds analyzed for each condition. Plates were completely covered with aluminum paper to assure darkness and then placed in an incubator at 28 °C (MIR-254-PE Cooled Incubator, PHC Europe B.V., Netherlands, 2023). The duration of the experiment was 5 days (including day 0 when the seeds were placed and day 4 when sprouts were removed). The humidity of the Petri dish was maintained by adding 2 ml of water or salt solutions on day 1 and day 3. At the end of the evaluation, the samples were placed immediately in liquid nitrogen and stored in the ultra-freezer. The frozen samples were processed into a fine powder by using pestle and mortar with liquid nitrogen. The samples were stored in ultra-freezer (-80 °C) until further analyses.

### Germination rate, length, weight and osmotic potential

The number of germinated seeds was counted in a 24 h interval while the length of the sprouts was measured on the last day (Day 4) through image analysis using the ImageJ program [31]. Total weight from sprouts from each petri plate was measured on the last day of the essay before storage. Growth rate (GR) was calculated with the following formula:

GR = (Final length/days).

While the osmotic potential (Ѱµ) was measured using 100 mg of fresh powder from each treatment using a freezing-point depression osmometer (Digital Osmometer, Roebling, Berlin) at 25 ± 1 °C [32].

### Analysis of ATP concentrations

Determination of ATP concentration was performed following a modified protocol [33]. ATP was extracted from 200 mg of stored powder, using 100 µl of pre-cooled methanol-water (1:1, v/v). Additionally, 50 µl of the internal standard (IS) solution (35 ng/mL N-acetylglucosamine, SIGMA-ALDRICH, Massachusetts, USA, 1975) was prepared as a control. After vortexing for 2 min, the samples were centrifuged at 12,500 x g for 15 min. A 100 µL of supernatant were analyzed using High-Performance Liquid Chromatography – Mass Spectrometry (HPLC-MS) at the Metabolomic and Proteomic Laboratory (ACTI - Murcia University, Spain). ATP was measured using an ATP patron (SIGMA-ALDRICH, Massachusetts, USA, 1975). The ATP concentration (ng g^− 1^) was used to calculate the ATP per sprout for a visually enhanced and more accurate measure.

### Analysis of minerals and nutrients

Macro- and micronutrient concentrations were determined using the freeze dried powders. The Inductively Coupled Plasma-Optical Emission Spectrometry (ICP-OES) analyses were conducted using a Thermo ICAP 6500 Duo instrument (Thermo Fisher Scientific, Waltham, MA, USA) as described by Nicolas-Espinosa et al. [7]. The nutrient concentrations were expressed as mg 100 g^− 1^ DW for macronutrients and mg g^− 1^ DW for micronutrients.

### Enzymatic assay for antioxidant activity

Antioxidant enzymes were extracted from 300 mg of the stored powder, using 50 mM potassium phosphate buffer (pH 7.8), containing 0.1 mM Na_2_–EDTA and 1% insoluble polyvinylpolypyrrolidone (PVPP). The 100 mM and control conditions were selected to simplify the interpretation and management of the results. This extract was used to determine the activity of the enzyme catalase (CAT) and oxidative damage to lipids [34]. The same extract with an added 10 mM β-mercaptoethanol was used for glutathione reductase (GR), or with 4 mM ascorbic acid for ascorbate peroxidase (APX) [35]. The supernatants were stored at − 20 °C for subsequent enzymatic assays.

Catalase (CAT) activity was measured by the disappearance of H_2_O_2_ as described by Aebi [36]. The extract was mixed with 50 mM potassium phosphate buffer (pH 7.0) and 10.6 mM H_2_O_2,_ monitoring the change in absorbance at 240 nm every 30 s for 3 min.

The oxidation rate of ascorbate was measured using ascorbate peroxidase (APX) activity as described by Amako et al. [37] and Nakano et al. [38]. The extract was mixed with 50 mM potassium phosphate buffer (pH 7.0) containing 0.1 mM Na_2_–EDTA, and 0.5 mM ascorbic acid. The addition of H_2_O_2_ was used to start the reaction and the decrease in absorbance at 290 nm was recorded every 30 s for 3 min.

Glutathione reductase (GR) activity was determined as described by Carlberg et al. [39] and Foyer et at [40]. The reaction mixture contained 0.1 M HEPES (pH 7.8), 1 mM Na_2_–EDTA, 3 mM MgCl_2_, 0.5 mM oxidized glutathione, and the enzyme extract. Finally, 0.2 mM NADPH was added. The rate of NADPH oxidation was monitored by the decrease in absorbance at 340 nm every 30 s for 2 min.

Bradford method [41] was used to determine the mg of protein in order to normalize the measures. The results of antioxidant activities (APX, GR and CAT) were expressed as µmol reactive min^− 1^ mg^− 1^ protein. Three biological and three technical replicates of the control and 100 mM treatments samples were analyzed.

### Lipid oxidative damage

Lipid peroxidation (LP) was determined using the extract from the antioxidant activity assay and determined as described by Minotti et al. [42]. The reaction was performed by mixing the extract with a reaction mixture containing 20% trichloroacetic acid (TCA), 0.5% 2-thiobarbituric acid (TBA), 0.01% butyl hydroxytoluene (HBT) and 0.25 N HCl, and incubated at 95ºC for 30 min. After stopping the reaction in ice and centrifuging the samples (10000 *x g*, 10 min), the supernatant was used for spectrophotometric reading at 535 nm. The calibration curve was made using serial malondialdehyde (MDA) concentration from 0 to 10 µM. The results were expressed in µmol MDA mg^− 1^ protein.

### Statistical analyses

Statistical analyses and visual presentation were conducted using Origin (Pro) (Version 2021 software package by OriginLab Corporation, Northampton, MA, USA). Significant differences among the values of all the parameters were determined using a one-way ANOVA with post hoc Tukey test and a statistical significance at *p* ≤ 0.05.

## Results

### Germinability and osmotic adjustment

**Fig. 1:**
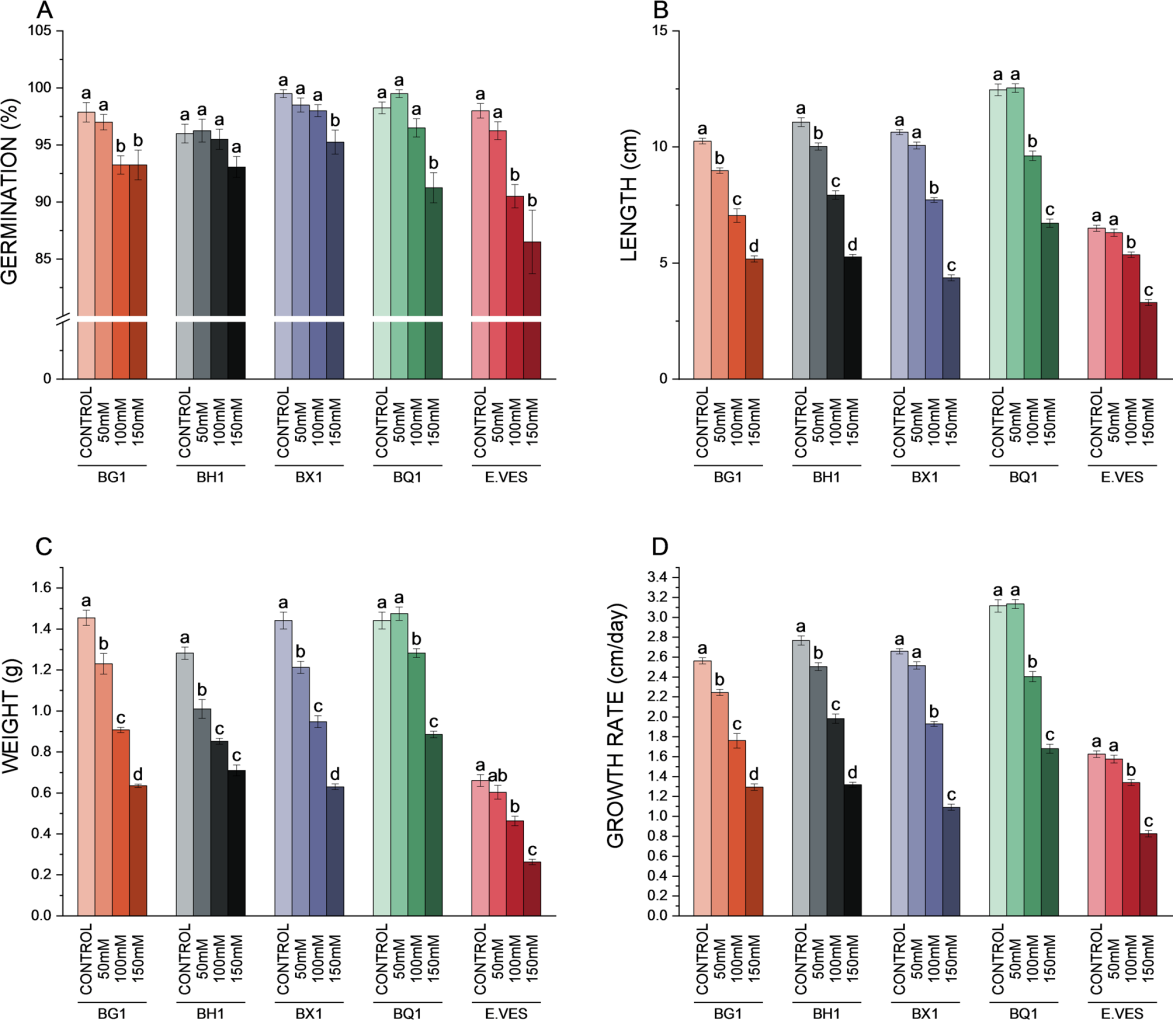
Physiological parameters in broccoli cultivars BG1, BH1, BX1 and BQ1, as well as *E. vesicaria* under control, 50 mM, 100 mM, and 150 mM NaCl conditions. (**A**) Germination rates (*n* = 100). (**B**) Sprout length (*n* = 6). (**C**) Sprout weight (*n* = 6). (**D**) Growth rate (*n* = 6). Different letters indicate significant differences according to one-way ANOVA followed by a *post hoc* Tukey´s test (*p* < 0.05) between treatments within the same species. Each bar represents the mean ± SE

Germination rates were above 90% in all broccoli cultivars across all treatments (Fig. 1A). No significant differences in germination rates were observed between treatments in the broccoli cultivars, except for BQ1 and BX1, where a significant decrease was observed under 150 mM treatment (Fig. 1A) and BG1, where the significant decrease was also observed under 100 mM. *E. vesicaria* showed a significantly greater decrease in germinability under the 100 mM and 150 mM treatments (Fig. 1A), compared to the broccoli cultivars. A direct correlation between an increase in salinity and a reduction in sprout length, weight and growth rate was observed in all varieties (Fig. 1B, C, D and Fig. sup.1). In the case of BX1, BQ1 and *E. vesicaria*, no significant differences were observed under control and 50 mM NaCl conditions for length and growth rate (Fig. 1B, D). Additionally, only BQ1 showed no weight differences under control and 50 mM NaCl conditions (Fig. 1C). Moreover, BQ1 exhibited the highest values for length, weight, and growth rate under all treatment conditions.

**Fig. 2:**
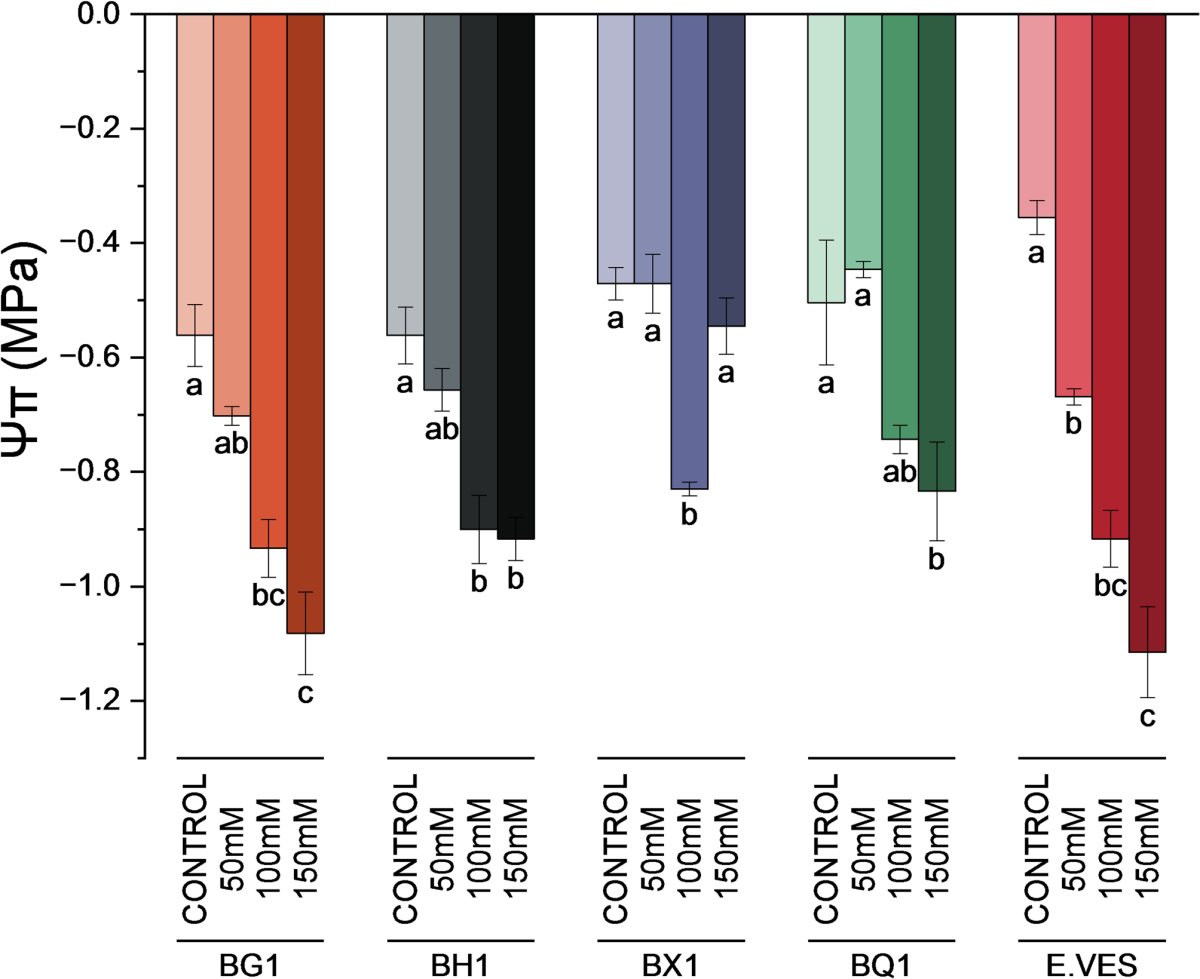
Osmotic potential value measured in BG1, BH1, BX1 and BQ1 broccoli cultivars and *E. vesicaria* under control, 50 mM, 100 mM, and 150 mM NaCl conditions. Different letters indicate significant differences determined by one-way ANOVA followed by Tukey´s *post hoc* test (*p* < 0.05) between treatments within the same species. Each bar represents the mean ± SE (*n* = 3)

In addition, when we analyzed the osmotic potential of sprouts, a significant decrease was observed in both species and cultivars across treatments (Fig. 2). BH1 and BQ1, showed no significant differences between the 100 mM and 150 mM NaCl conditions, but both values were lower compared to the control. In BX1, the lowest value was observed at 100 mM, but osmotic potential at 150 mM was similar to the control.

Although no statistical analysis was performed between species and cultivars, noticeable differences were observed under the 150 mM salinity condition, specifically BG1 and *E. vesicaria* showed the lowest values, while BX1 showed the highest.

### Analysis of ATP concentrations

**Fig. 3:**
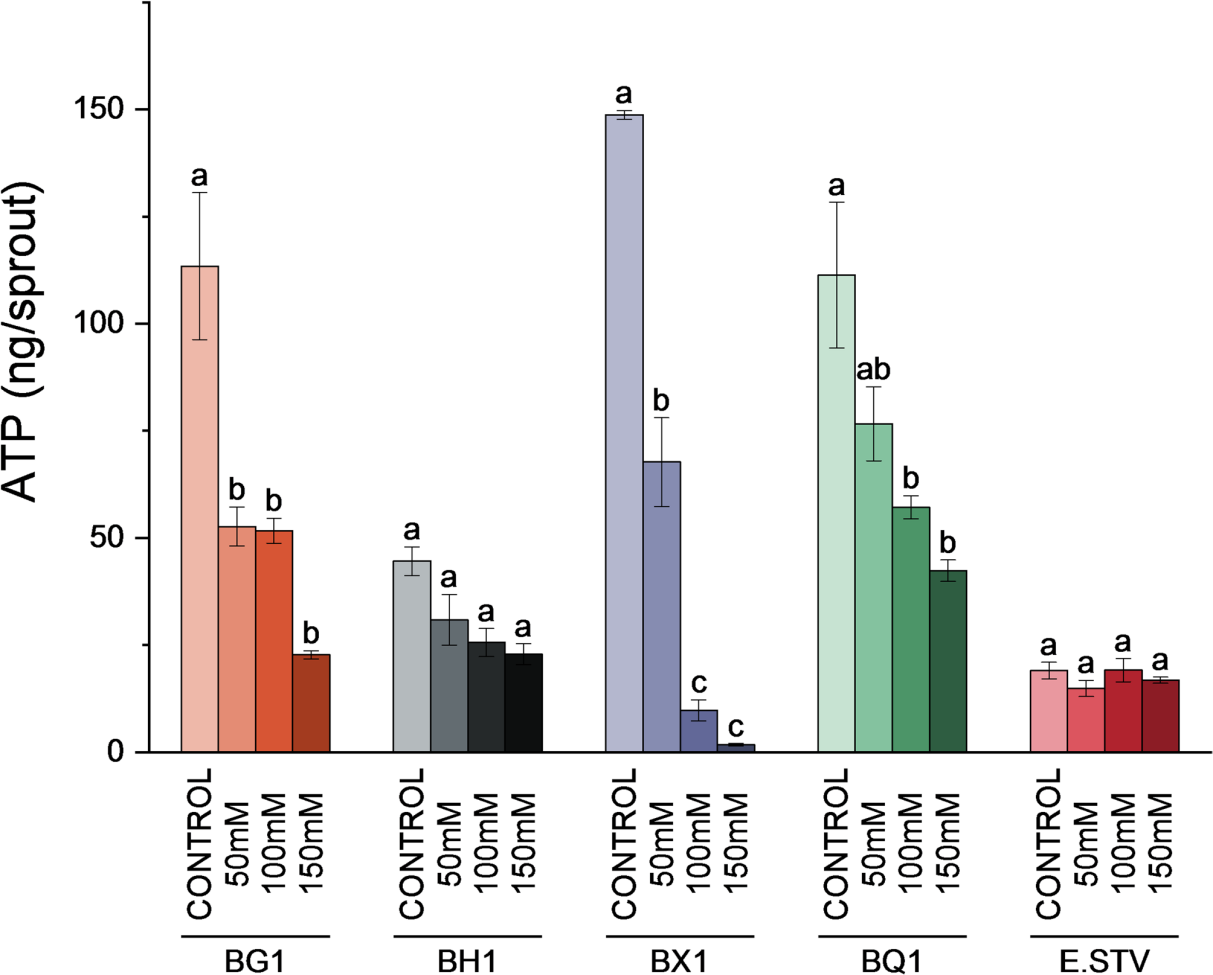
ATP concentration in ng per sprouts in BG1, BH1, BX1 and BQ1 broccoli cultivars and *E. vesicaria* under control, 50 mM, 100 mM, and 150 mM NaCl conditions. Different letters indicate significant differences according to one-way ANOVA followed by Tukey´s *post hoc* test (*p* < 0.05) between treatments within the same species. Each bar represents the mean ± SE (*n* = 3)

ATP concentration per sprout showed a significant reduction in some of the broccoli cultivars under salinity (BG1, BX1 and BQ1) but no differences were observed in BH1 and *E. vesicaria* (Fig. 3). Both species also showed the lowest value of ATP concentration in control conditions. The largest difference between treatments was observed in the BX1 cultivar, which exhibited the biggest value in control condition and the lowest in the 150 mM treatment.

### Analysis of minerals and nutrients

**Fig. 4:**
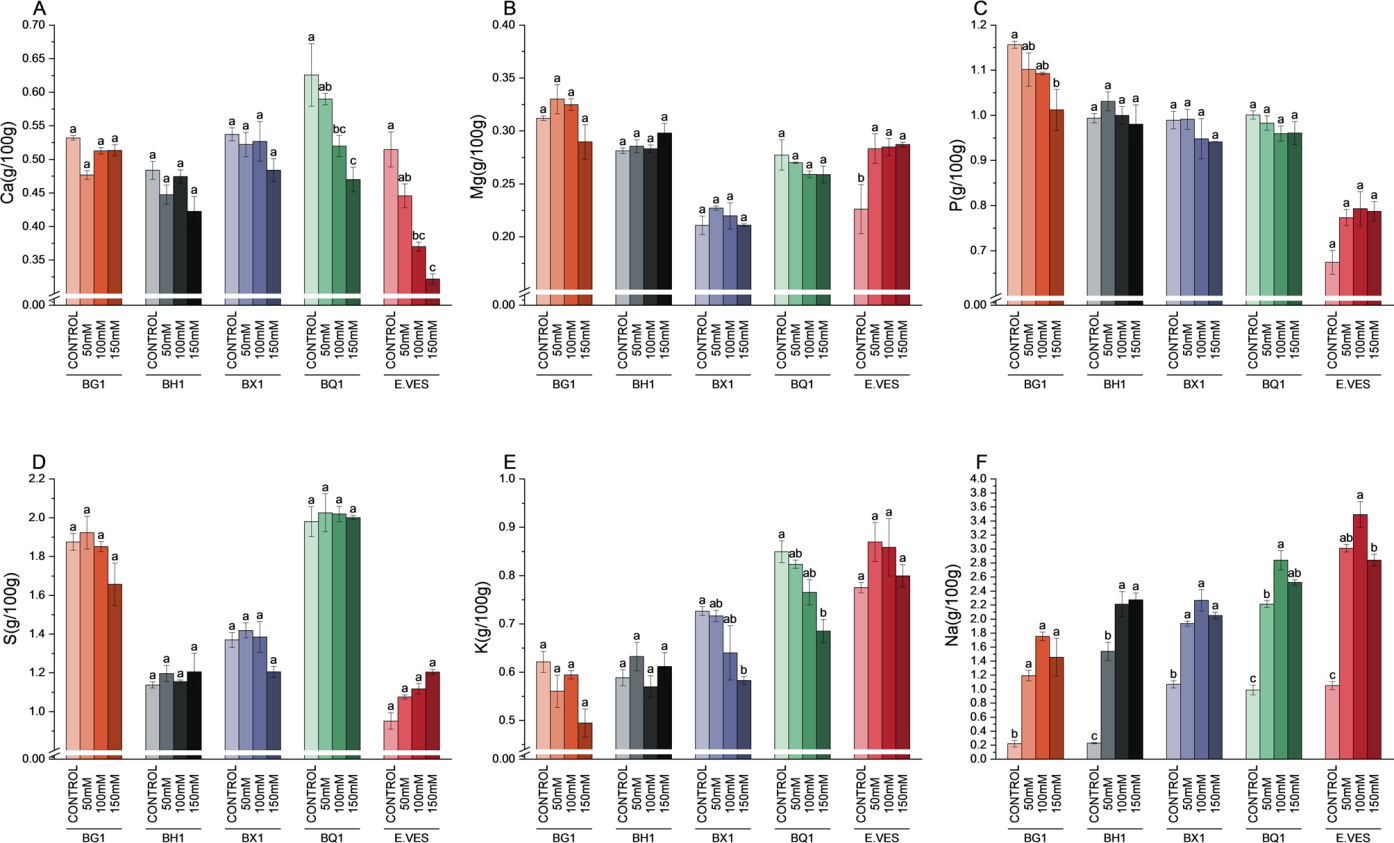
Macronutrient concentration (g/100 g) in BG1, BH1, BX1 and BQ1 broccoli cultivars and *E. vesicaria* under control, 50 mM, 100 mM, and 150 mM conditions. Different letters indicate significant differences according to one-way ANOVA followed by a *post hoc* Tukey´s test (*p* < 0.05) between treatments within the same species. Each bar represents the mean ± SE (*n* = 3)

**Fig. 5:**
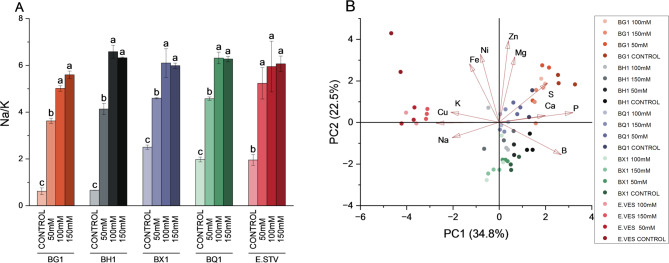
(**A**) Na/K ratio in BG1, BH1, BX1 and BQ1 broccoli cultivars and *E. vesicaria* expressed in molar values under control, 50 mM, 100 mM, and 150 mM conditions. (**B**) Principal Component Analysis (PCA) of macro- and micronutrients in BG1, BH1, BX1 and BQ1 broccoli cultivars and *E. vesicaria* under control, 50 mM, 100 mM, and 150 mM conditions. Different letters indicate significant differences according to one-way ANOVA followed by a *post hoc* Tukey´s test (*p* < 0.05) between treatments within the same species. Each bar represents the mean ± SE (*n* = 3)

Alterations in mineral composition were observed across species, cultivars, and specific minerals analyzed. A significant decrease in Calcium (Ca) was observed in BQ1 under a progressive increase of salinity concentration, but a similar trend was also observed among the other species (Fig. 4A). *E. vesicaria* exhibited the lowest Ca level under salinity stress while Magnesium (Mg) concentration was significantly increased. BG1 under saline conditions showed a decrease in Phosphorous (P) (Fig. 4B) while *E. vesicaria* had the lowest values under all conditions. Notably, sulfur (S) concentration did not change with increasing salinity; however, each cultivar showed difference in intrinsic S levels. BG1 and BQ1 were grouped together with higher S concentrations, while BH1 and BX1 exhibited the lowest levels. A decrease in potassium (K) concentration was observed in BX1 and BQ1 cultivars under salinity (Fig. 4E) but the highest K levels in all treatments were observed in BX1, *E. vesicaria*, and BQ1 under control conditions. Na concentrations increased under salinity (Fig. 4F); however, concentrations reimaned unchanged with increasing salinity. BX1, BQ1 and *E. vesicaria* showed the highest Na values under control conditions. The Na/K ratio increased under salinity treatments in all cultivars and species (Fig. 5A); nevertheless, no differences were observed between the different cultivars and *E. vesicaria*. PCA analysis showed a clear differentiation between the broccoli varieties and *E. vesicaria* on the horizontal axis when the macro- and micronutrient sprout compositions were analysed (Fig. 5B), particularly under the different conditions. Moreover, distinct groups were observed: BG1 and BQ1 clustered together, while BH1 and BX1 formed a separate group.

### Enzyme assay and lipid oxidative damage

**Fig. 6:**
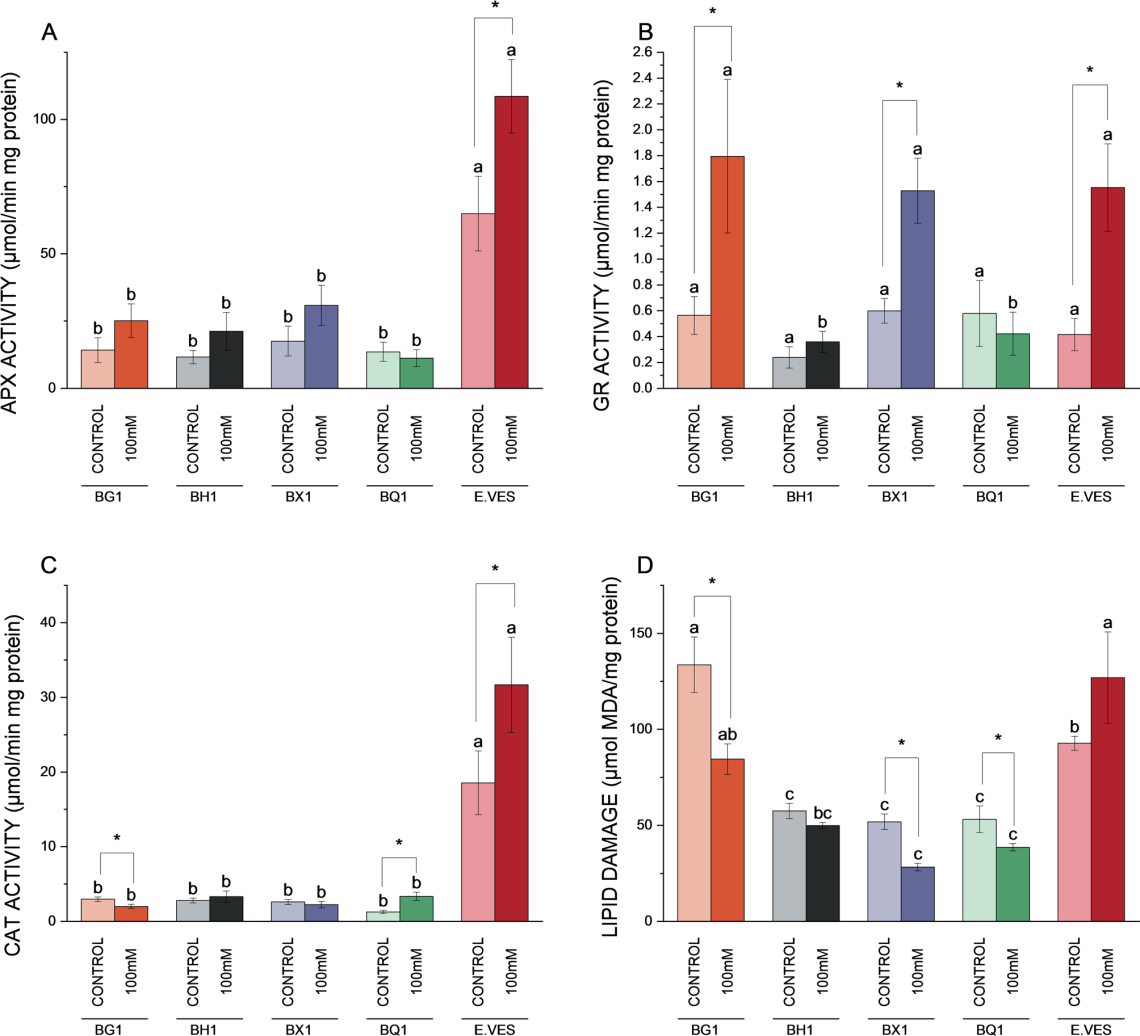
Enzymatic activity and lipid damage measure in BG1, BH1, BX1 and BQ1 broccoli cultivars and *E. vesicaria* under control and 100 mM conditions. (**A**) Ascorbate peroxidase activity. (**B**) Glutathione reductase activity. (**C**) Catalase activity. (**D**) Lipid oxidative damage. Different letters indicate significant differences according to one-way ANOVA followed by a *post hoc* Tukey´s test (*p* < 0.05) between species within same treatment. Asterisks (*) indicate significant differences between treatments within the same cultivar or species according to one-way ANOVA followed by a *post hoc* Tukey´s test (*p* < 0.05). Each bar represents the mean ± SE (*n* = 3)

For enzyme activity and lipid oxidative damage, the 100 mM and control conditions were selected to simplify the interpretation and management of the results. Ascorbate peroxidase (APX) activity showed a significant increase in *E. vesicaria* under saline conditions (Fig. 6A). The highest activity was observed in *E. vesicaria* under both control and salinity conditions. Glutathione reductase (GR) activity increased in BG1, BX1 and *E. vesicaria* under salinity conditions (Fig. 6B). All broccoli cultivars presented a low catalase (CAT) activity, which was only altered by salinity in BG1 and BQ1. *E. vesicaria* showed a notably high basal CAT activity under control conditions and showed a more notable significant increase in CAT activity under salinity conditions (Fig. 6C). Lipid oxidative damage showed a significant decrease in all broccoli cultivars, except for BH1 and *E. vesicaria*, where no changes were observed under salinity conditions (Fig. 6D).

## Discussion

### Germinability

Seeds are complex structures equipped with the essential stored compounds to support germination and early seed growth. These intrinsic characteristics, however, vary significantly among species and cultivars, influencing their ability to withstand environmental stresses, such as salinity. In the current study, physiological, biochemical, and molecular parameters under control and salinity conditions during seed germination of four different broccoli cultivars were investigated to determined salt tolerance. These results were later compared with salt-tolerant species *E. vesicaria*. Our results indicate that germination rates were primarily determined by genotype, with salinity concentration playing a secondary role. This is supported by the relatively stable germination rates observed among the broccoli cultivars, with no significant changes under 150 mM NaCl, except for BG1. In contrast, *E. vesicaria* exhibited a significant and more pronounced reduction in germination rate under both 100 mM and 150 mM NaCl treatments. This significant decrease observed in *E. vesicaria* has also been observed in the germination rate of different species under salinity stress within the *Brassicaceae* family. Studies conducted on different crops have shown similar evidence regarding the abiotic stress related tolerance that was exhibited at the germination. For example, while tomato presented moderate salt tolerance during later developmental stages, this species demonstrated high sensitivity to salinity during germination, as evidenced by a reduction in germination rates under salt stress conditions [43]. Also, Bybordi [44] observed that *Brassica napus* exhibited a significant decline in germination at 150–200 mM NaCl, which is consistent with other studies conducted on radish and beetroot [45, 46]. Three cultivars of broccoli have been observed with a decline in germination under 80–100 mM NaCl [47]. Similarly, 100 mM NaCl significantly decreased germination rates in broccoli sprouts [48]. Therefore, these previous studies indicated that salinity concentrations above 80 mM negatively affected seed germination in *Brassicas*, but our results did not show significant differences in germination rates under 150 mM salinity conditions. This could be due to prior selection of salt-tolerance at mature stages among pre-commercial broccoli cultivars as it was previously reported [49]. Despite the non-significant effect of salinity on germinability, a decline in other physiological parameters under salinity conditions was observed. All broccoli cultivars exhibited a reduced length and growth rate with increasing salinity concentration. However, BX1, *E. vesicaria*, and particularly BQ1, demonstrated greater tolerance under 50 mM salt concentration based on growth rate, plant length, and biomass weight. A similar reduction of length and weight in broccoli sprouts treated with NaCl concentrations higher than 80 mM was also observed by Tian et al. [50].

Although *Eruca vesicaria* subsp. *sativa* is widely recognized as a highly salt-tolerant species, our study showed that its tolerance was not observed at the germination stage. This suggests that some of the tolerance mechanisms may not be active during germination. A similar phenomenon has been observed in other halophyte species, such as *Cakile maritima*, which fails to germinate under high salinity (200 mM) despite its ability to grow at much higher concentrations [51]. This pattern may represent an adaptive strategy to prevent germination in unfavorable environments, such as seawater-exposed conditions, where high salinity levels have a detrimental impact on sprout establishment and survival.

### Osmotic adjustment and ATP

Salinity is a major abiotic stress that disrupts germination and sprout development by imposing osmotic stress, ion toxicity, and nutrient imbalances. To mitigate the damage caused by salinity, plant cells typically synthesize and accumulate osmoprotectant molecules to regulate the osmotic potential of the cells, thus enhancing water absorption under these saline conditions. The reduction in osmotic potential is a common adaptive response of plants to cope with salt stress and has been previously reported in broccoli, where the accumulation of compatible osmolytes, has been associated with salinity tolerance in broccoli cultivars [52]. A progressive accumulation of osmolytes appeared to be associated with the decrease in osmotic potential caused by the increase in salt concentration, particularly in BG1 and *E. vesicaria*. In contrast, in BH1, BX1 and BQ1, osmolyte accumulation was triggered when the seeds were exposed to 100 mM salinity and maintained at a similar concentration when salinity increased to 150 mM, except for BX1. This suggest that BH1 and BQ1 employ a threshold-dependent response to salinity, where osmolyte accumulation is triggered only when stress reaches a critical level (100–150 mM). This reduces unnecessary energy expenditure during mild stress (e.g., 50 mM), preserving resources for higher stress levels. Furthermore, BX1 disrupted ATP reserve management under salt stress, exhibiting a marked reduction in sprout ATP content at higher salt concentrations (100 mM and 150 mM). This could be related to its inability to synthesize compatible osmolytes due to an energy-saving mechanism, leading to a lack of osmotic potential adjustments in these plants. Moreover, this specific cultivar demonstrated the poorest performance under salinity stress conditions, indicating the essential role of osmotic potential adjustments in salinity tolerance, which is directly related to energy management and ATP usage.

### Mineral nutrients

Changes in Ca concentrations in response to increased salinity were observed exclusively in the BQ1 cultivar and *Eruca vesicaria*. However, the Ca concentrations in BQ1 remained comparable to those measured in the other broccoli cultivars across all salinity levels, whereas *E. vesicaria* consistently exhibited significantly lower Ca concentrations than the broccoli cultivars under all salinity conditions indicating a direct effect of salinity in this species. Ca has been reported to mitigate the adverse effects of salinity in plants, as salinity conditions reduce Ca uptake by displacing it from the cell membrane or disrupting membrane function, leading to increased Na accumulation in leaves and impairing K/Na selectivity [53]. Similar findings have been observed in other halophyte species, where calcium supplementation alleviated salinity-induced damage, as Na directly affected cell wall properties and plasma membrane function through Na/Ca displacement [54]. In this context, maintaining adequate Ca levels is critical for salinity tolerance, which was observed exclusively in the broccoli cultivars under salinity conditions, suggesting enhanced mechanisms to prevent Ca displacement. BQ1 exhibited higher calcium levels under control conditions, suggesting this cultivar has inherently elevated seed calcium content. This trait may serve as an adaptive mechanism to mitigate salinity-induced stress without expending energy to transport calcium in order to maintain membrane stability. P reduction under salinity was observed exclusively in the BG1 cultivar, with *E. vesicaria* again exhibiting the lowest values. This suggests these two species show reduced absorption or impaired mobility, as certain phosphate transporters are sensitive to salt, hindering P uptake and its mobilization from internal reservoirs [55]. A reduction in K levels under salinity conditions was observed only in the BX1 and BQ1 cultivars, which, along with *E. vesicaria*, exhibited the highest K concentrations under salinity. This reduction is understandable as Na and K show similar chemical properties indicating that both share channels reducing K transport or absorption [56]. BQ1 showed the highest growth rate and maintained the highest K concentration at 150 mM NaCl, suggesting a potential correlation between growth performance, salinity tolerance, and K accumulation. Given the importance of K homeostasis in salinity tolerance, seed K levels may serve as an indicator of salt stress resilience. Notably, BQ1 exhibited the highest K concentration in seeds. With regards to Na, the broccoli cultivars with the highest physiological measures in salinity (BX1 and BQ1) along with *E. vesicaria* showed the highest concentration of Na in control conditions, indicating that the levels of Na in these seeds are the highest. A clear relation between seed exposure to higher Na levels in seed and a better tolerance to salinity is evident, but also supported by previous studies where high Na concentrations in seed has been observed in halophytes species [57, 58]. Another significant phenomenon is that all sprouts exhibited an increase in Na concentration upon exposure to 50 mM NaCl; however, this intracellular Na concentration remained stable despite further increases in exogenous salinity (100 mM and 150 mM). This suggests that seeds have a threshold for intracellular Na accumulation, likely regulated by Na compartmentalization within the vacuole. This threshold appeared to be reached at 50 mM, which could be reasonable considering that these seeds are only five days old and still undergoing development. At this stage, vacuolar structures and their associated transport mechanisms may be predominantly functional in the cotyledons, as these tissues are already differentiated. This compartmentalization mechanism effectively mitigates ionic toxicity, stress and cellular damage. However, as salinity increases, vacuolar sequestration becomes insufficient, suggesting the activation of alternative adaptive mechanisms. Given the absence of significant changes in overall Na concentration within the sprouts, these compensatory mechanisms may involve Na efflux through ATP-dependent transporters, highlighting the critical role of energy allocation in salinity tolerance.

Based on the observed changes in mineral composition, BQ1 is the one showing a distinct pattern. BQ1 displays the highest values of Ca, K and Na in seed, all of which are key elements associated with salinity stress tolerance. An early exposure to Na along with an enhanced concentration of Ca and K can generate the perfect environment to trigger the mechanism to prevent excessive oxidative damage under salinity. Some of these mechanisms have already been discussed such as Na sequestration within the vacuole, exclusion of excess Na via selective ion transporters as SOS1 or HKT transporters as well as K retention through efficient K channels to maintain enzymatic function and osmotic balance [59]. The combined ability of BQ1 to manage Na and K levels minimizes ion toxicity and osmotic imbalance supporting sustained growth and metabolism under salinity. However, *E. vesicaria* might rely on more limited or less efficient mechanisms, such as early vacuolar sequestration of Na, which could limit its overall tolerance to saline environments.

### Antioxidant activity

Antioxidants, including ascorbate and glutathione, play a critical role in enhancing plant defense mechanisms against oxidative stress. These antioxidant levels are directly related to the antioxidant enzymatic activities of APX, GR and CAT activity that are part of the oxidative stress response. A significant increase in APX activity under salinity and non saline conditions was observed only in *E. vesicaria.* GR activity showed a significant increase in BG1, BX1 and *E. vesicaria* under salinity conditions. Regarding CAT activity, broccoli cultivars showed low activity in both treatments while *E. vesicaria* had the highest values, being 4 times higher in control and 10 times higher in salinity. These results indicate how *E. vesicaria* exhibited the highest and strongest antioxidant enzymatic response. BQ1 and BH1 are the ones that showed no differences between treatments indicating no oxidative damage response through antioxidant enzymes routes. Exposure to salinity conditions is reported to increase antioxidant enzymes activity such as APX, GR and CAT in plants as observed in mungbean [60] under 100 mM. The same has been reported in other species such as Djulis [61]. Therefore, the absence of changes in these activities under salinity conditions may indicate an alternative response pathway that does not involve antioxidant enzymes or the sprout not experiencing oxidative stress. The results suggest that the ion homeostasis mechanisms in these cultivars exhibit superior functionality compared to others, thereby mitigating oxidative stress since the Na/K ratio remains consistent across all broccoli cultivars. BQ1 and BH1 appear to exhibit more efficient sodium compartmentalization. Consequently, the SOS1-NHX1 system in these two cultivars may have higher expression levels or more effective isoforms. To gain a clearer understanding of this process, lipid oxidative damage is a key parameter to consider. All broccoli cultivars except for BH1 showed a decrease in lipid oxidation, indicating that the various antioxidant mechanisms at play are effectively preventing oxidative damage under salinity conditions. *E. vesicaria* did not show this reduction; instead, an increase was observed. This implies how BQ1 and BH1, which showed no significant increase in antioxidant enzyme activity under salinity, displayed both low lipid oxidative damage, while *E. vesicaria*, despite its high antioxidant activity, failed to mitigate oxidative damage effectively. BQ1 and BH1 may depend on non-enzymatic antioxidant systems or it can be attributed to an efficient sodium compartmentalization mechanism to mitigate oxidative damage resulting in a reduced necessity for enzymatic pathway activation. This could be facilitated by pre-synthesized antioxidant reservoirs stored in seeds or the presence of upregulated Na transporters. Plants significantly activate the aspartate-glutathione pathways to synthesis these non-enzymatic antioxidants involved in detoxify ROS up to tolerable levels under salinity conditions [62]. Moreover, observing that even the cultivars that did not show changes in enzyme activity still exhibited no increase in lipid oxidative damage provides evidence of an alternative mechanism. These alternative mechanisms may be closely related to cellular energy balance. The antioxidant system requires energy in order to function properly and to synthesize antioxidant molecules such as ascorbate and glutathione. ATP values were the lowest in BH1 and no changes were observed under salinity conditions, which was also observed in *E. vesicaria*,. This suggests that all mobilized or synthesized ATP is rapidly utilized in antioxidant defense mechanisms or in the activation of membrane ATPases, enhancing the capacity for Na and K transport. This facilitates the sequestration of Na into the vacuole. In BH1, the ATP mobilization and usage can be more related to antioxidant molecules biosynthesis and Na sequestration to prevent ROS damages, as no changes in enzymatic activity were observed. Among the other cultivars, we observe higher ATP pools in the seed and higher ATP use, indicating that a larger ATP availability can allow multiple responses. This explains why BQ1 showed better germination and sprout growth under high levels of salinity with no changes in enzymatic activity. Futhermore, it can be inferred that BQ1 may be using its large ATP pool to trigger alternative mechanism, some already described such as ion efflux mechanisms to alleviate ion toxicity, with Na⁺ toxicity being particularly significant and the maintenance of ion homeostasis, particularly K⁺ and Ca^2+^ homeostasis. This is important since Ca also plays an important role in processes that preserve the structural and functional integrity of plant membranes avoiding ROS damage [63]. The threshold-triggered osmolyte accumulation strategy is equally important as it minimise energy wastage under low stress conditions (50 mM) while enabling a robust response under severe stress (100–150 mM). The majority of these mechanisms are ATP-dependent, underscoring the critical role of intracellular energy levels in maintaining optimal performance of these mechanisms [23]. Therefore, ATP levels may represent a key determinant of the differential responses profiles observed in sprouts. A larger ATP pool, and more importantly, a well-regulated ATP utilization, can be considered critical tolerance traits in early-stage sprouts, as they enable prolonged and more diverse physiological responses over time.

## Conclusion

The results revealed that all broccoli cultivars exhibited higher germination rate and superior physiological performance compared to *E. vesicaria* at germination, with the highest performance observed in the BQ1 cultivar. These findings suggest that different mechanisms are activated in each development stage across cultivars and species to mitigate salinity-induced stress. These mechanisms include solute accumulation, enhancement of antioxidant activity alongside alternative strategies such as Na⁺ sequestration and exclusion, K⁺ transport, and overall mineral homeostasis. All these processes are energy-dependent, relying on ATP availability. Thus, ATP availability emerges as a key factor defining the salinity response profile of each cultivar. These profiles differentiate cultivars in terms of salinity tolerance, revealing specific physiological strategies. BG1 efficiently utilizes a moderate ATP pool to maintain performance under moderate salinity (50–100 mM). BH1 exhibits limited stress responses due to low ATP availability. Although BX1 possesses the largest ATP reservoir, it fails to regulate ATP allocation effectively, compromising osmotic regulation. In contrast, BQ1 maintains a balanced ATP utilization strategy, enabling the activation of a broader range of adaptive mechanisms. Meanwhile, *E. vesicaria* showed limitation to prevent oxidative damage despite its physiological responses. Overall, BQ1 integrates multiple protective strategies to mitigate, prevent, and tolerate salinity-induced stress in sprouts, achieving the highest physiological performance and outperforming the enzymatic responses observed in *E. vesicaria.*

## Electronic supplementary material

Below is the link to the electronic supplementary material.

**Supplementary Material 1**: **Additional file 1**. Seed images. Images of the seeds of the four broccoli cultivars and *E. vesicaria* in day 1 and day 4 in control, 50 mM, 100 mM and 150 mM conditions.

## Data Availability

Data is provided within the manuscript.
