# Supplementary figures and images for: Comparative adaptations of high-tolerant species and broccoli cultivars to salinity stress during germination and early development stages
Source: BMC Plant Biol. 2025 May 27;25:706. doi: 10.1186/s12870-025-06723-3 (PMC12108041; doi:10.1186/s12870-025-06723-3)

|       | CONTROL                                                                             | 50 mM                                                                               | 100 mM                                                                              | 150 mM                                                                              |
|-------|-------------------------------------------------------------------------------------|-------------------------------------------------------------------------------------|-------------------------------------------------------------------------------------|-------------------------------------------------------------------------------------|
| BG1   | 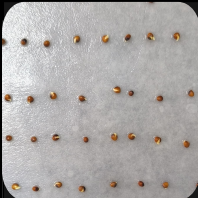   | 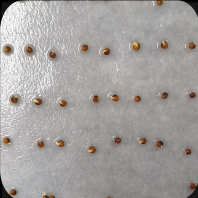   | 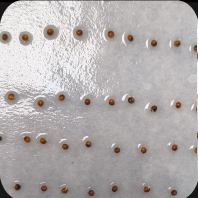   | 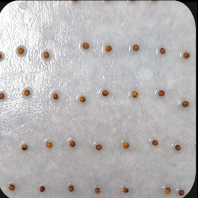   |
| DAY 1 | 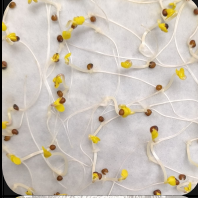   | 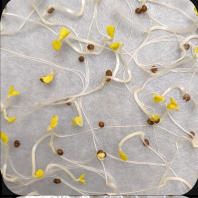   | 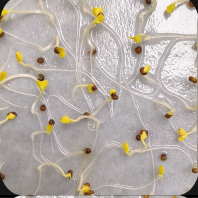   | 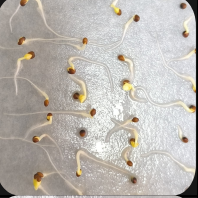   |
| DAY 4 | 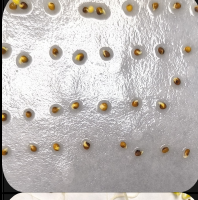   | 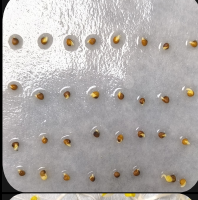   | 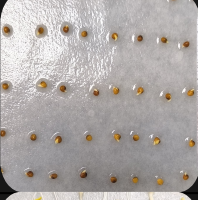   | 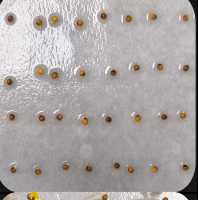   |
| BH1   | 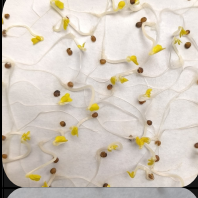   | 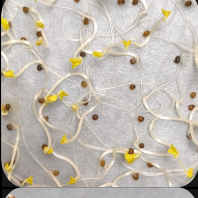   | 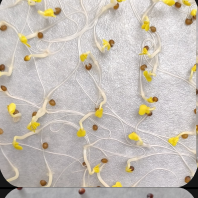   | 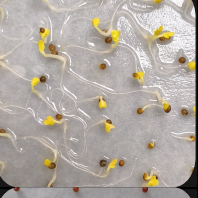   |
| DAY 1 | 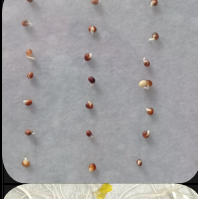  | 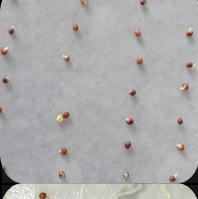  | 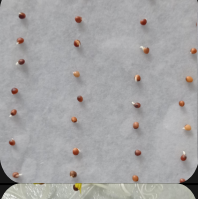  | 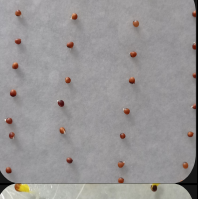  |
| DAY 4 | 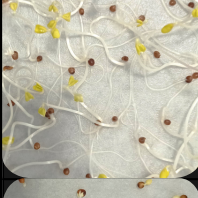 | 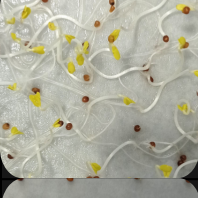 | 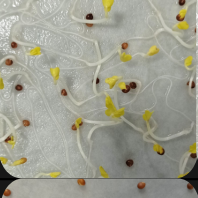 | 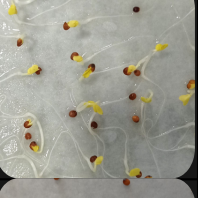 |
| BX1   | 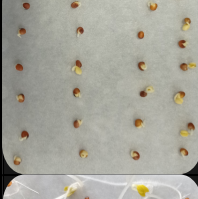 | 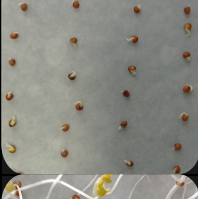 | 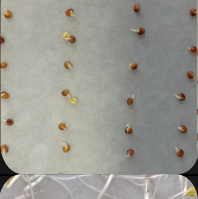 | 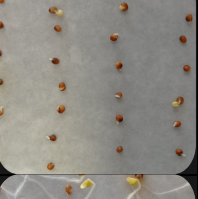 |
| DAY 1 | 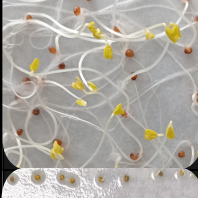 | 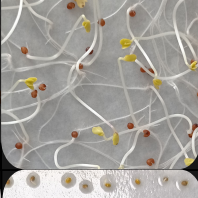 | 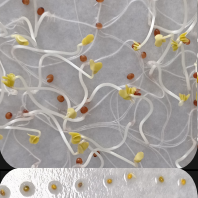 | 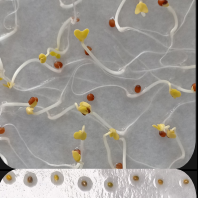 |
| DAY 4 | 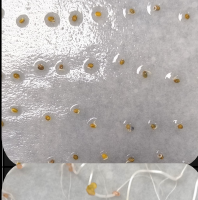 | 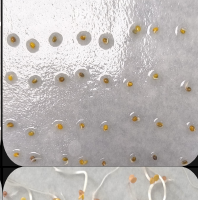 | 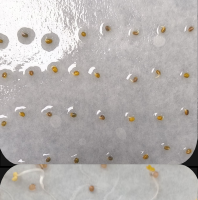 | 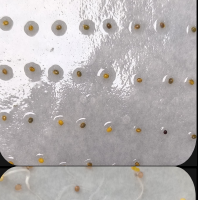 |
| BQ1   | 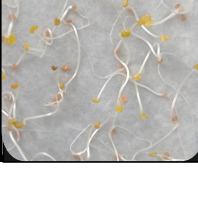 | 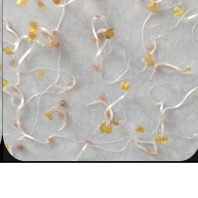 | 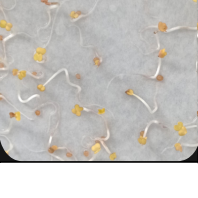 | 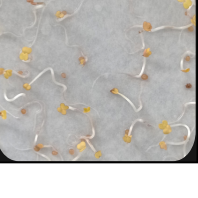 |
| DAY 1 | 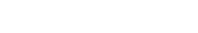 | 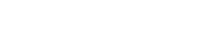 | 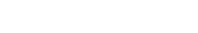 | 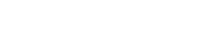 |
| DAY 4 |  |  |  |  |
| E.STV |  |  |  |  |
| DAY 1 |  |  |  |  |
| DAY 4 |  |  |  |  |

Supplement: Supplementary file 1 — Supplementary Material 1: Additional file 1. Seed images. Images of the seeds of the four broccoli cultivars and E. vesicaria in day 1 and day 4 in control, 50 mM, 100 mM and 150 mM conditions. [file 12870_2025_6723_MOESM1_ESM.pdf]
